# Essay Read before the Iowa State Dental Association

**Published:** 1865-10

**Authors:** G. W. Nichols

**Affiliations:** Davenport, Iowa


### ESSAY READ BEFORE THE IOWA STATE DENTAL ASSOCIATION.

BY G. W. NICHOLS, DAVENPORT, IOWA.

GENTLEMEN :—Having been appointed at the last regular meeting of our society as an essayist for this occasion—no doubt more in the spirit of encouragement, than otherwise, I chose for my subject "Mechanical Dentistry,"— not that I propose to teach my Dental brethren anything in regard to that, which, if they are Dentists, they must be already practically acquainted with the various *methods* of mechanical substitution; or, that I would arrogate to myself the knowledge of certain principles, which, though they may not have occurred to many, are certainly open to all — but I thought I would take advantage of my appointment, to give expression to a few general observations and reflections which have presented themselves from time to time. First, I have observed a deep, wide spread, and I fear, a growing tendency to sacrifice anything in the shape of a natural tooth that presents itself for *replacement.* This arises, no doubt, in the first place from a too general lack of knowledge on the part of the patronizing public, causing them often to sadly underestimate the truly great value of their own natural organs of mastication, and altogether *over*estimate the comparative comfort and use of artificial ones. This renders them careless and inattentive to the decay of their teeth, and backward about expending a few dollars upon their preservation. It is common for the Dentist to have good teeth presented for extraction—teeth, that with a little care might yet be made to serve a lifetime—and be told, as he expostulates with his patient, endeavoring to have him or her see the matter in its true light—that "no," those teeth shall never trouble me any more, "take them out, or I will get some one else to," and out they come. What wonder, when this same patient is imbued with the idea that a full artificial set can be had at about the price of a pair of boots; having no adequate conception, and often even, never having heard anything in regard to those great functions of the animal economy—Mastication and Digestion.

Besides this, there are, I am pained to say, many practitioners in this profession, Dentists in name, but charlatans, swindlers and thieves in practice, who foster this ignorance on the part of their patients—men, or shapes of men, who, having learned, or picked up somewhere the way — not the Art, for few have mastered that, but *the way* to stick bits o baked Porcelain on an uncouth plate, having no skill, and lacking every requisite necessary to the composition of a Dentist—thinking of nothing beyond dollars, and caring for nothing beyond self. Devils in human form, for I can call them nothing less — that go about from place to place (for few locations are peopled with such midnight ignorance as to sustain them for any length of time) seeking whom they may devour. Stuffing unprepared cavities with mercurial paste; removing tartar with quicklime and aquafortis, and wherever an opportunity can be found, or forced, removing teeth promiscuously and inserting in their stead, those patent, inimitable, compound, and double prominent, $13 sets. Now, this is all wrong. In the first place, every scholar in our common schools, ought to be early and particularly instructed in regard to the vital importance of the Dental apparatus; its liability to decay; and the care and watchfulness necessary to its preservation. They should also, be shown something in regard to the difficulties and capabilities of Dental practice, that they may not only be led to a just appreciation and timely consultation of Dental skill, but that they may also have within themselves a criterion established, by which they can discriminate between the man of real professional ability and his tinsel prototype, the vender of quack nostrums and mere impudence.

On the other hand, a Dentist should be compelled to obtain by actual knowledge, and known capacity, either the diploma of a Dental faculty, or a legal certificate, signed by not less than three, long experienced and well known dentists. On these, as a foundation, could be reared a superstructure of living prices and real excellence. And then, having established the foundations of Mechanical Dentistry, in the patronage of an enlightened and appreciative public, and in the services of intelligent, competent and honorable operators, we should find that the only indication for artificial substitution was where, all conditions and circumstances considered, the *actual benefit* of such procedure outweighed *every other* consideration. Here we may extract with a clear conscience, and firm hand, furnishing our patron with the best substitute that our ingenuity can devise.

This brings me to another part of my subject, that partially owing to the present peculiar construction of block teeth, whose ridged arch admits of but little adjustment, and partially from a lack of care, taste, or a feeling of inadequate remuneration, nine-tenths of, at least, the cheaper class of Dental substitutes are but miserable abortions. Teeth they are, to be sure, they could not well be less. The operator is furnished them by the manufacturer; but what selection, and what arrangement! Look at the Dental arch. How is it imitated? Instead of a wedge like form, terminating in a short curve, we frequently see glaring upon us from the harmless mouths of many a poor victim, horrible semicircles of white porcelain, reminding us more of the Idol teeth of savage barbarians, than the scientific structure of professional men. Let this thing end, let us have a greater variety of curves in blocks, and a *great deal more care* on the part of those who use them.

Let the Dental aspirant for professional perfectability go to the mouths of his patients where all the teeth remain, and see how nature builds her arch, as to color, size, and last, though not least of all, expression. Here a great many of our best operators have fallen into an egregious error. They assume that artificial teeth should be set upon the plate with perfect regularity, for if Nature had a chance to do her best, that is the way she would set them. Were this a true assumption, yet we know that Nature has not the chance to do her best, she finds something continually to thwart and interfere with her designs, and if we hope to attain to her peculiar harmonizing of the contour and texture of the face, with the substance and arrangement of the teeth, we must study *her as she is.* If we do this, if we set teeth as she sets them, seldom, if ever regular, with this cusp in and that cusp out, this central true with the arch, and that slightly turned, this tooth this way, and that the other, *turned in the mouth* until the effect is right, we shall soon hear less of that "crowning horror"—false teeth. The contrast between the face and teeth will be lessened, and all join together as harmonious parts of one expressive whole. It is here, as elsewhere, that "the proper study of mankind is man." He that notes nature closely will not be likely to place the pearls of blooming sixteen, in the sallow face of eighty; nor, try to hoop the jaw with a round strap of teeth, but, rather with a wholesome and large consideration, adopt that course which his enlightened observation, best judgment, and the *highest interest of his patient,* sanctions.

